# Editorial: Interfacing Humans and Machines for Rehabilitation and Assistive Devices

**DOI:** 10.3389/frobt.2021.796431

**Published:** 2022-01-03

**Authors:** Carlos A. Cifuentes, Jan F. Veneman, Eduardo Rocon, Carlos Rodriguez-Guerrero

**Affiliations:** ^1^ Department of Biomedical Engineering, Colombian School of Engineering Julio Garavito, Bogota, Colombia; ^2^ Hocoma AG, Volketswil, Switzerland; ^3^ Centro de Automática y Robótica, Consejo Superior de Investigaciones Científicas-Universidad Politécnica de Madrid (CSIC-UPM), Madrid, Spain; ^4^ Robotics and Multibody Mechanics Research Group, Department of Mechanical Engineering, Vrije Universiteit Brussel and Flanders Make, Brussels, Belgium

**Keywords:** prosthetics and orthotics, exoskeletons, human-robot interfaces, brain-machine interfaces, sensors and actuators for human-robot interaction

## Introduction

Currently, around 10*%* of the world’s population, or roughly 650 million people, live with some type of disability. In countries with life expectancies over 70 + years, people spend on average about 8 years, or 11.5 percent of their lifetime, living with disabilities (Disabled World (2021). Di, 2021).

In response to this need scientists from different fields, together with engineers and clinicians have been working on developing robotic solutions for a wide variety of rehabilitation and assistive scenarios. Robotic exoskeletons are now commonly found tools used at neurorehabilitation centers, treating stroke and spinal cord injury survivors. Occupational exoskeletons are now alleviating a big part of the harmful body loading, responsible for widely common musculoskeletal disorders found in industrial settings. Bionic prostheses are now making their way through the markets and getting attention by the social security systems around the world and will most likely become widely adopted in the near future. All the aforementioned technologies require interfacing humans and robots to assure a safe and efficient cognitive and physical interaction. Here, the interface refers to any hardware or software link that connects two dissimilar systems: humans and robots.

**FIGURE 1:**
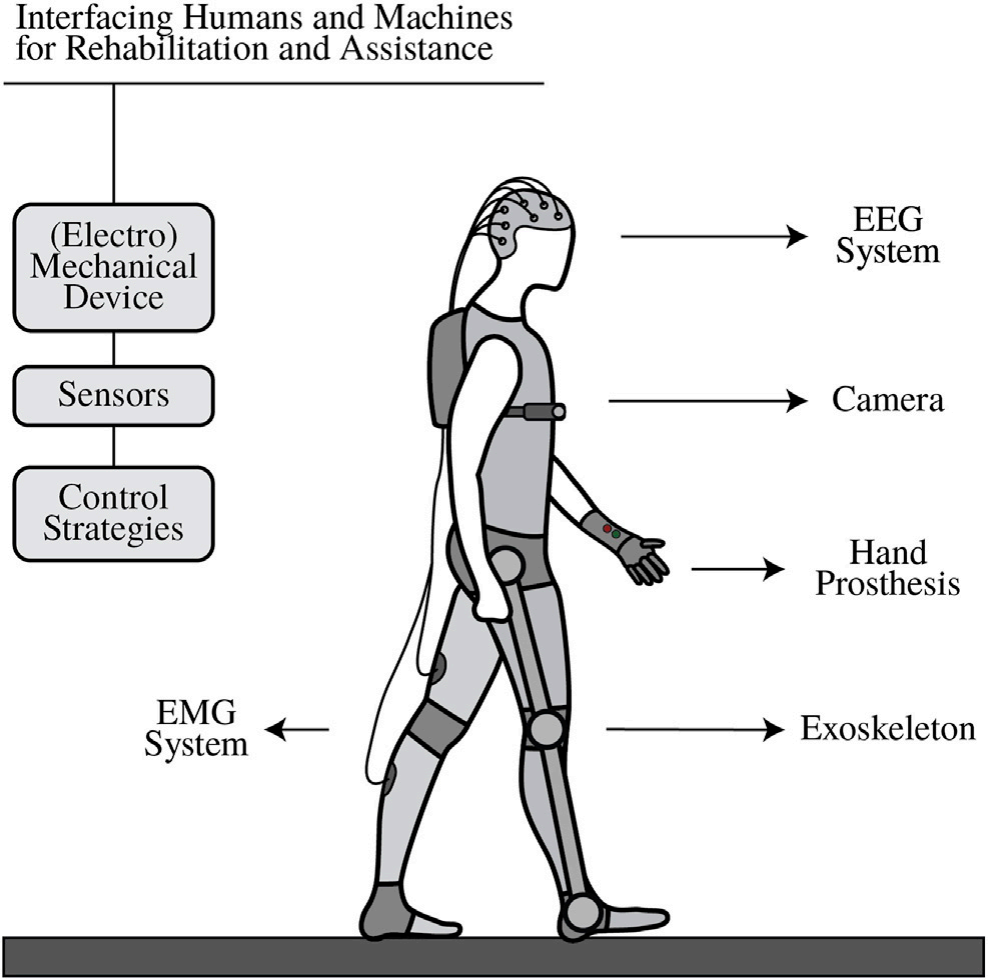
Interfacing humans and machines for rehabilitation and assistive devices.

The topic “*Interfacing Humans and Machines for Rehabilitation and Assistive Devices*” was opened to gather professionals and researchers from various backgrounds and discuss the pertinence and feasibility of new human-robot interfaces in the field of rehabilitation and assistance. The community’s outstanding response to the call led to 18 contributions by 97 different authors that address the requirements and challenges of implementing and deploying rehabilitation and assistive robotics (see Figure 1). The contributions proposed new control and modeling strategies for orthotic and prosthetic devices (for both upper and lower limbs), explored methodologies to detect human intention, and assess quantitative and qualitative measurements of the behavior and outcomes when interfacing humans and machines.

The research topic comprises promising state-of-the-art developments in a broad spectrum of devices. There are many ways to address and classify rehabilitation and assistive devices, not forgetting the differences between them. A rough categorization could be whether 1) they are worn or integrated on the body, or 2) they are external tools either handled by the user or by the therapist. The first group can then be subdivided into whether 1) they replace (or add) a body structure, what are known as prostheses, or 2) they support body functions by supporting existing body structures, known as orthoses.

Prosthetics can derive from different technological sources. One that has gained significant attention is innovations in desktop 3D printers and open-source designs that lead to creating body-powered, 3D printed prosthetic hands. Based on the additive deposition of material in a layer-by-layer manner to construct parts from a 3D computer-aided design (CAD) model, these devices have disrupted the field of rehabilitation and assistive devices and are every day more available to be implemented as functional low-cost alternatives. However, there are fundamental interfacing issues that need to be addressed for long-term usage. In this case, Cabibihan et al. evaluated them to benefit children with congenital missing limbs and the war-wounded.

On the other side, there are more advanced electro-mechanical prostheses, also called bionic arms or hands. These devices have been designed to approximate the natural limb in both form and function. They have continuously achieved a better range of functional grip, power, and precision. However, they do not match the dexterity of natural hands yet, and several challenges arise from the human-machine interaction (HMI) comprising sensing, control, and actuation. Accurate and efficient interaction includes the implementation of biological signals such as EMG recordings from the residual limb and EEG and various clever control strategies (Brinton et al.); (Frolov et al.).

In the field of orthoses, which are mechanical structures that correct, support, restore and enhance the function of a part of the body instead of replacing it, the contributions here presented focused on their robotic counterparts: the robotic exoskeletons. In this case, exoskeletons for different parts of the body and varying configurations of material are studied.

Upper limb exoskeletons are commonly used in the industry by workers during long-hour tasks and in robotic-assisted rehabilitation therapies, in both cases to support repetitive movements. The use of soft technology has gained significant attention in upper limbs as compliant interaction with the subject favors its purpose. The detection of the human intention of motion is fundamental to controlling these robotic devices to assist humans according to their needs. Similar to strategies presented with orthotic devices, novel approaches for detecting hand motion intention and controlling the exoskeletons are based on EMG signals (Islam and Bai). In addition to the development of robotic kinematics and control, the study of proper methods to design physical HMI plays a fundamental role in the comfort and usability of the device, as presented by Perry et al. Just like some upper limb exoskeletons, occupational back-support exoskeletons are every day, more commonly used to mitigate work-related pain. Poliero et al. evaluated the impact of carrying activities on lower-back loading than lifting to select different assistive strategies.

The exoskeletons that have been more intensely studied are, without a doubt, lower limb exoskeletons. The actuation system implemented in them is one of the essential factors in their design as it generally determines the performance, efficiency, and portability. There are mainly three types of actuators used in modern exoskeletons: 1) electrical motors, 2) pneumatic actuators, and 3) hydraulic actuators. Even though some researchers choose pneumatic or hydraulic actuators due to their higher power/weight ratio or better compliance, most exoskeletons use electric motors due to their precision and ease of control. Therefore, the analysis of components as gearboxes, elastic elements, and transmission systems is critical in developing lower limb exoskeletons (García et al.). There are many commercially available examples with various technologies implemented in the market. However, researchers are constantly in the quest for new, more natural ways of controlling these devices. From bio-inspired controllers 1) based on motor primitives (Nunes et al.) or 2) developed to allow dynamic standing balance (Fasola et al.) to approaches that naturally decodes a neuromuscular surrogate (Karunakaran et al.), contributions explore the development of strategies to match a healthy gait pattern better. (Laschowski et al.), for example, introduced an environment recognition system to improve the control of robotic lower-limb exoskeletons and prostheses during human locomotion.

As the new exoskeletons are developed and tested in the market and the research centers, the need for standardized assessment measures and characterization increases. Methods such as analyzing the dynamic margins of stability during robot-assisted gait are a way to robustly and objectively measure such devices’ performance (Ramanujam et al.). However, other more clinically related parameters that could help to assess the impact of their use could be 1) to determine the number of training sessions necessary to achieve adequate exoskeletal-assisted walking skills and velocity milestones (through the implementation of well-known walking tests as presented by Hong et al. or 2) to keep track of adverse events and associated risks when performing robot-assisted gait training (Bessler et al.). Since there is a wide range of possibilities to correctly assesses lower limb exoskeletons, a benchmarking framework becomes more and more necessary. Benchmarking wearable robots is then a vital task to quantify both the technical performance of the devices and the physical impact they have on the users (Torricelli et al.).

Additionally, somewhere between prostheses and orthoses are the Supernumerary Robotic Limbs (SRL), also called extra theses. They consist of additional robotic body parts (e.g., limbs and fingers) to augment the user’s abilities. SRL function together with an intact musculoskeletal system, but add an utterly functional body structure, not a replacement, but still as a structure and not as a support of an existing structure.

They have been initially proposed for industrial purposes and differ from exoskeletons, as they do not request any joint-to-joint alignment. The analysis of compensatory movements performed by this SRL determines the future usability of this type of system moving forward into assistive applications, as presented by Rossero et al.

Even though this first big group of devices worn or integrated on the body is the most renowned device in the field, rehabilitation, and assistive technology, either handled by the user or by the therapist, offer patients with disabilities other opportunities.

To this second group belong devices for functional gait compensation such as crutches, walkers, and wheelchairs, and each of them represents a whole area of research and development. Among them, canes are the devices more commonly used to increase gait stability. A simple single-point cane may prevent or reduce falls in patients with imbalance. Similar to orthosis, robotic counterparts for each of the devices mentioned above exist. Smart walkers, robotic wheelchairs, and robotic cane embrace the same challenge of interfacing with humans for optimal performance (Trujillo-León et al.).

Other rehabilitation robots, not necessarily in the field of orthosis, are also used in training setups. End-effector-based systems are robotic systems that are only attached to the distal segments of the limbs and belong to this group. They include, for example, cable-driven motion support robots. Compared to exoskeletons, these systems require more minor adjustments to each patient. However, they need the motion of all adjacent segments to be inferred using mechanical models or additional sensors, such as inertial units presented by Passon et al.

Interfacing humans and machines for rehabilitation and assistive devices evidently encompasses many possible devices and design choices that directly affect the living conditions of people who have suffered from motor impairments or amputations. In pursuit of practical functionality, these solutions require robust interfaces that allow natural and compliant control. Possibilities are endless, and the contributions gathered in this topic invite professionals and researchers from various backgrounds to collaborate and share promising developments where humans and machines are interfaced in rehabilitation or assistive environments. The editors and authors of this affluent and evolving research topic believe that this space, with all its different rehabilitation and assistive devices, can mutually inspire developers in their quest for a better quality of life.
